# Electrostatic Theory of the Acidity of the Solution in the Lumina of Viruses and Virus-Like Particles

**DOI:** 10.1021/acs.jpcb.2c08604

**Published:** 2023-03-07

**Authors:** H. J. Muhren, Paul van der Schoot

**Affiliations:** Soft Matter and Biological Physics, Department of Applied Physics and Science Education, Eindhoven University of Technology, Postbus 513, 5600 MB Eindhoven, The Netherlands

## Abstract

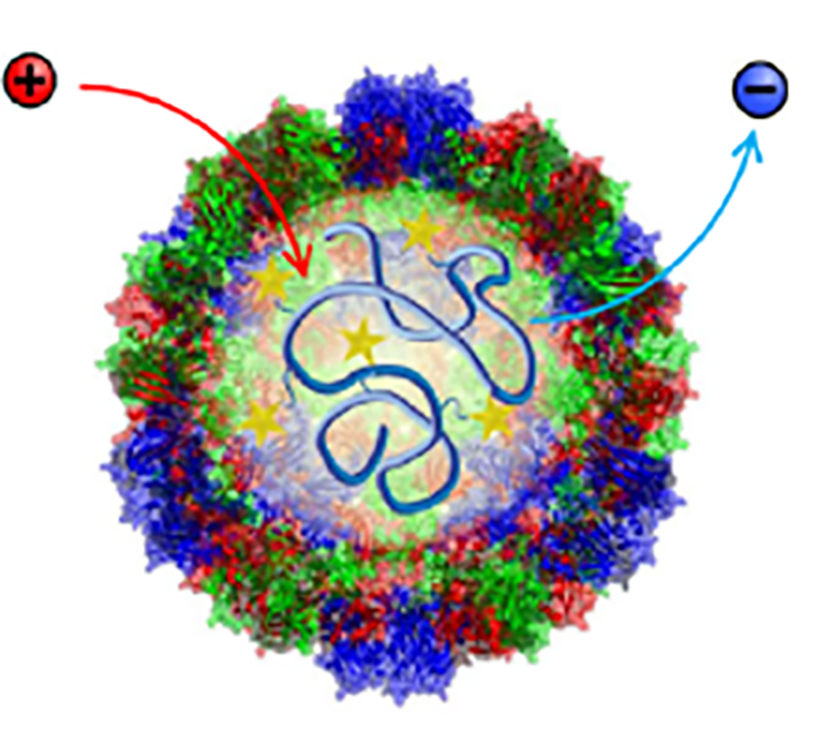

Recently, Maassen et al. measured an appreciable pH difference between the bulk solution and the solution in the lumen of virus-like particles, self-assembled in an aqueous buffer solution containing the coat proteins of a simple plant virus and polyanions (Maassen, S. J.; et al. *Small***2018**, *14*, 1802081). They attribute this to the Donnan effect, caused by an imbalance between the number of negative charges on the encapsulated polyelectrolyte molecules and the number of positive charges on the RNA binding domains of the coat proteins that make up the virus shell or capsid. By applying Poisson–Boltzmann theory, we confirm this conclusion and show that simple Donnan theory is accurate even for the smallest of viruses and virus-like particles. This, in part, is due to the additional screening caused by the presence of a large number of immobile charges in the cavity of the shell. The presence of a net charge on the outer surface of the capsid we find in practice to not have a large effect on the pH shift. Hence, Donnan theory can indeed be applied to connect the local pH and the amount of encapsulated material. The large shifts up to a full pH unit that we predict must have consequences for applications of virus capsids as nanocontainers in bionanotechnology and artificial cell organelles.

## Introduction

The coassembly of the coat proteins of many simple viruses with their single-stranded (ss) RNA seems to be primarily driven by electrostatic interactions between the negatively charged genome and the positively charged, disordered RNA binding domains on the coat proteins.^1−4^ It is no surprise, then, that under appropriate solution conditions virus coat proteins spontaneously encapsulate not only homologous but also heterologous ssRNAs, synthetic polyanions, surface-functionalized nanoparticles, and so on.^5−11^ Interestingly, the coassembly does not necessarily produce virus-like particles of the same size or T number or even the same shape as that of the native virus. Size and shape selection seems to be controlled on the one hand by mass action and hence stoichiometry, and on the other by a compromise between the interaction between the coat proteins and that between the coat proteins and cargo.^8,12,13^ Virus coat proteins, such as that of Brome Mosaic Virus, Cowpea Chlorotic Mosaic Virus, and Hepatitis B Virus, can actually be made to self-assemble into shells in the absence of a negatively charged cargo by changing the acidity and/or salinity of the solution, removing the RNA binding domain of the coat proteins or chemically modifying this domain.^14−16^ The spontaneous assembly is in that case driven by hydrophobic and other types of attractive interaction, involving, e.g., ionic and hydrogen bonds.^17,18^

Because virus coat proteins are able to encapsulate molecular cargo, either spontaneously from solution or by attaching it chemically or physically to, e.g., the RNA binding domain of the protein, there is a significant interest in utilizing this property for application purposes in targeted drug delivery, tomography, controlled catalysis, and metamaterials.^19−22^ Recently, virus-based artificial organelles have been suggested as a viable route to be used in living cells for therapeutic purposes, restoring or even adding cellular activity.^23^ These artificial organelles contain catalytically active particles such as enzymes. The reason that encapsulated catalytic particles can actively process substrates present in the solution, is that the capsid shell is permeable to these molecules but only if they are not too large in size. If too large, substrates cannot diffuse through the pores in the shell that include ones with a diameter as large as a few nanometers.^24^ The semipermeability of capsids, in particular, that of plant viruses, has been known for some time.^25^

Catalytic activity, in general, is strongly pH and ionic strength-dependent, suggesting that the control of the physicochemical conditions of the solution in the lumen of the virus capsid or any other type of proteinaceous shell used for the same purpose must be of paramount importance.^26^ Interestingly, so far this issue seems to have met with relatively little attention in the artificial organelle community,^23^ even though recent experiments by Maassen et al. demonstrate that the pH in the lumen of a virus-like particle can be much lower than that of the bulk solution.^27^ It is interesting to mention that the lumina of carboxysomes, which are proteinaceous shells that act as organelles in bacteria, also seem to have a lower pH than the bulk solution both in vitro and in vivo.^28^ The reason that the acidity and ionic composition of the lumen of a protein shell can be much different than that of the bulk solution is the presence of a net immobile charge in that lumen, that is, a net charge not associated with translationally mobile species that can freely diffuse in and out of the shell, and the result of what is commonly known as the Donnan effect.^29^ This effect has long been known to cause pH gradients across lipid membranes in the context of vesicles.^30^

In the experiments of Maassen et al.,^27^ the coat protein of cowpea chlorotic mottle virus (CCMV) and a 7.5 kDa random copolymer of styrenesulfonate and pH-sensitive fluorescent fluorescein methacrylate monomers spontaneously assemble in virus-like particles under a wide range of solutions pHs. The authors use the ratio of two excitation peaks to obtain the relation between the pH in free solution and that in the lumen. For pHs spanning values between 6.0 and 8.0, the pH inside the virus-like particle turns out to be about 0.4 pH units lower than that outside of it, that is, in the bulk solution. A simple Donnan theory put forward by the authors, presuming a uniform distribution of immobile charges in the lumen and ignoring both the presence of the protein shell holding the polyanionic cargo and the presence of a net charge on the outer surface of it, actually explains this observation. In the present context, Donnan theory presumes (i) local charge neutrality, meaning in this case that both the capsid lumen and the bulk solution outside the virus are electroneutral, and (ii) equal chemical potentials of the mobile ionic species in- and outside of the shells.^31,32^ Typically, the well-known expressions for the electrochemical potentials of the charged mobile ionic species valid in dilute solution are used for the latter. Standard chemical potentials and the effects of nonideality of the solution, insofar that these can be described by activity coefficients, can be absorbed in the Donnan potential, which actually acts as a Lagrange multiplier enforcing local charge neutrality. This implies that the theory should be valid even if the ionic solution does not behave ideally.^27^

According to the theory, the dimensionless Donnan potential ϕ in the capsid lumen, defined as the Donnan potential energy scaled to the thermal energy, obeys the simple relation^33,34^
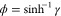
1where the quantity γ ≡ Δρ/2ρ_s_ is defined in terms of the difference Δρ = ρ_+_^i^ – ρ_–_^i^ of the number densities of positive and negative immobile charges ρ_±_^i^, averaged over the volume of the lumen, and 2ρ_s_ the overall density of mobile ionic species in the bulk solution (tacitly presumed to be monovalent). This quantity is dominated by the concentrations of added salt and buffer of the assembly mixture, and explains why in practice the Donnan potential and also the pH differential across the protein shell is virtually independent of the acidity of the solution,^27,28^ unless some form of charge regulation takes place involving weakly ionic moieties on the cargo or the coat proteins themselves.^27,35^

The pH shift, defined as the difference ΔpH = pH_in_ – pH_out_ between the solution pH_in_ inside and pH_out_ outside of the particle, is proportional to the Donnan potential,
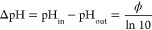
2which follows from the Boltzmann distribution that connects the concentrations of the mobile ionic species in- and outside of the shell. With a value of ΔpH ≃ −0.4 obtained from the experiments, eqs 1 and 2 suggest a value of γ ≃ −1.1. The ionic strength of the mobile ionic species present in the lumen is as a result of this almost 50 percent larger than that in the bulk solution, which is physiological and equal to 0.15 M.^27^ Computer simulations confirm that the mobile ionic content of the capsid lumen must be very different from that in the bulk solution.^36^

A negative value of γ implies that a larger number of negative polymer charges have been encapsulated than necessary to compensate for the positive charges on the RNA binding domains. This phenomenon is usually referred to as charge reversal or overcharging, and γ can therefore be seen as a measure for the degree of over- or undercharging.^37−40^ Taking as a radius for the lumen a range of 4–5 nm, an ionic strength of 0.15 M, and 600 positive charges on the RNA binding domains, we obtain for the relative overcharging −Δρ/ρ_+_^i^ a value corresponding to 8–15%. This translates to between 10 and 18 encapsulated chains.^27^ Conversely, we can use the fact that a large number of *T* = 3 viruses with a genome smaller than about 6000 nt seem to have an average degree of overcharging of +60%,^41^ and convert this number into a pH shift. According to Donnan theory, this degree of overcharging translates to a pH shift of −1, if we take the average of 10 or so positive charges per RNA binding domain of those viruses.^41^ Overall, relative degrees of overcharging ranging from −100% for empty capsid shells due to the absence of any charge compensation by immobile cargo because in that case ρ_–_^i^ = 0, to +800% for encapsulated high-molecular weight poly(styrenesulfonate) have been reported.^18^ This then suggests that, if we take eqs 1 and 2 at face value, there must be a large spread in pH shift in virus-like particles ranging in value from about −1 and +1 depending on the amount of encapsulated cargo, ionic strength, and so on.

Of course, these estimates hold only if Donnan theory, which presumes a spatially uniform electrochemical potential in the lumen of the protein shell, actually applies on the scale of *T* = 1 and 3 viruses. Such small viruses measure between 20 and 30 nm in the outer diameter, while their lumina are obviously even smaller than that, and, say, 10–20 nm wide.^42^ Typical electrostatic screening lengths are around one nanometer and any encapsulated polyanion should perhaps be expected to be concentrated primarily in the region where the RNA binding domains are located, which for CCMV for instance is estimated to be about three or four nanometers wide.^4,13,38^ Theoretically, the effect seems to be smaller for linear polyanions than for ssRNA-like randomly branched ones, and for *T* = 1 particles, this layer should extend almost to the center of the cavity of the capsid.^43,44^ One might attempt to construct a nonuniform Donnan theory, in a similar vein as was done by Odijk and Slok for densely packed double-stranded DNA in bacteriophages,^45^ and by Philipse in the somewhat different context of charged colloids in a gravitational field.^46^ In the present context of over- or undercharged virus-like particles such an enterprise would defeat our purpose, which is to obtain estimates from a simple theory that requires as few input parameters as possible, and that does not require taking recourse to numerical methods.

Even if the distribution of the immobile charges in the lumen is more or less uniform, as we expect it to be for *T* = 1 sized shells, we nevertheless think it is prudent to investigate under what conditions simple Donnan theory applies, and how accurate it actually is given that the mobile charges tend to not be uniformly distributed.^34^ We note also that within Donnan theory the presence of a net charge on the outer surface of the shell, which can be net positive or net negative depending on the type of virus and pH of the solution,^42^ does not affect the predicted pH shift. The reason is that for typical concentrations of coat protein up to a hundred μM in in vitro experiments and typical numbers of surface charges of perhaps a few hundred, the corresponding concentration of surface charges should remain much lower than the ionic strength of the buffer solution that typically is (near) physiological.

By applying Poisson–Boltzmann theory to model virus-like particles, we are able confirm that predictions for pH shifts obtained from Donnan theory are quite accurate also for the smallest, that is, the *T* = 1 sized particles, even for ionic strengths much below physiological. The reason is that in practice the overall concentration of immobile ionic species in the capsid lumen is very much larger than the ionic strength of the buffer solution, and because of this also that of the mobile ionic species. This gives rise to a smaller effective screening length in the capsid lumen, which may in fact also be inferred from earlier work on the electrostatics of soft particles.^34,47,48^ The impact of a surface charge is relatively minor, in part caused by the presence of the capsid shell that has a finite thickness. Interestingly, a net charge on the shell that has the same sign as the net immobile charge in the lumen potentially increases the accuracy of Donnan theory.

The remainder of this paper is organized as follows. First, in the section Theory, we formulate a Poisson–Boltzmann theory for a spherical charge distribution inside of a shell that has a net positive or negative surface charge on its outer surface. Following Lifson and others,^33,34,48,49^ we write the electrical potential in the cavity of the shell as the sum of a uniform and a position dependent part that we treat as a perturbation, where we identify the uniform part as the Donnan potential. We solve the equations analytically for the position-dependent potential (i) up to linear order in the perturbation in the cavity, (ii) exactly in the shell and (iii) at the level of a Debye–Hückel approximation in the outer region. Next, in the section Results and Discussion, we analyze our findings, the most important one being that under a wide range of conditions the correction to the pH shift obtained from Donnan theory, caused either by nonuniformity of the electrical potential or the presence of surface charges on the shell, is minor. Finally, we provide a summary of our findings in the section Discussion and Conclusions, and discuss how both Donnan theory and Poisson–Boltzmann theory can be amended to account for the effects of charge regulation and explain why in the experiments of Maassen and collaborators the pH in the capsid lumen becomes constant below a pH of 6.^27^

## Theory

**Figure 1:**
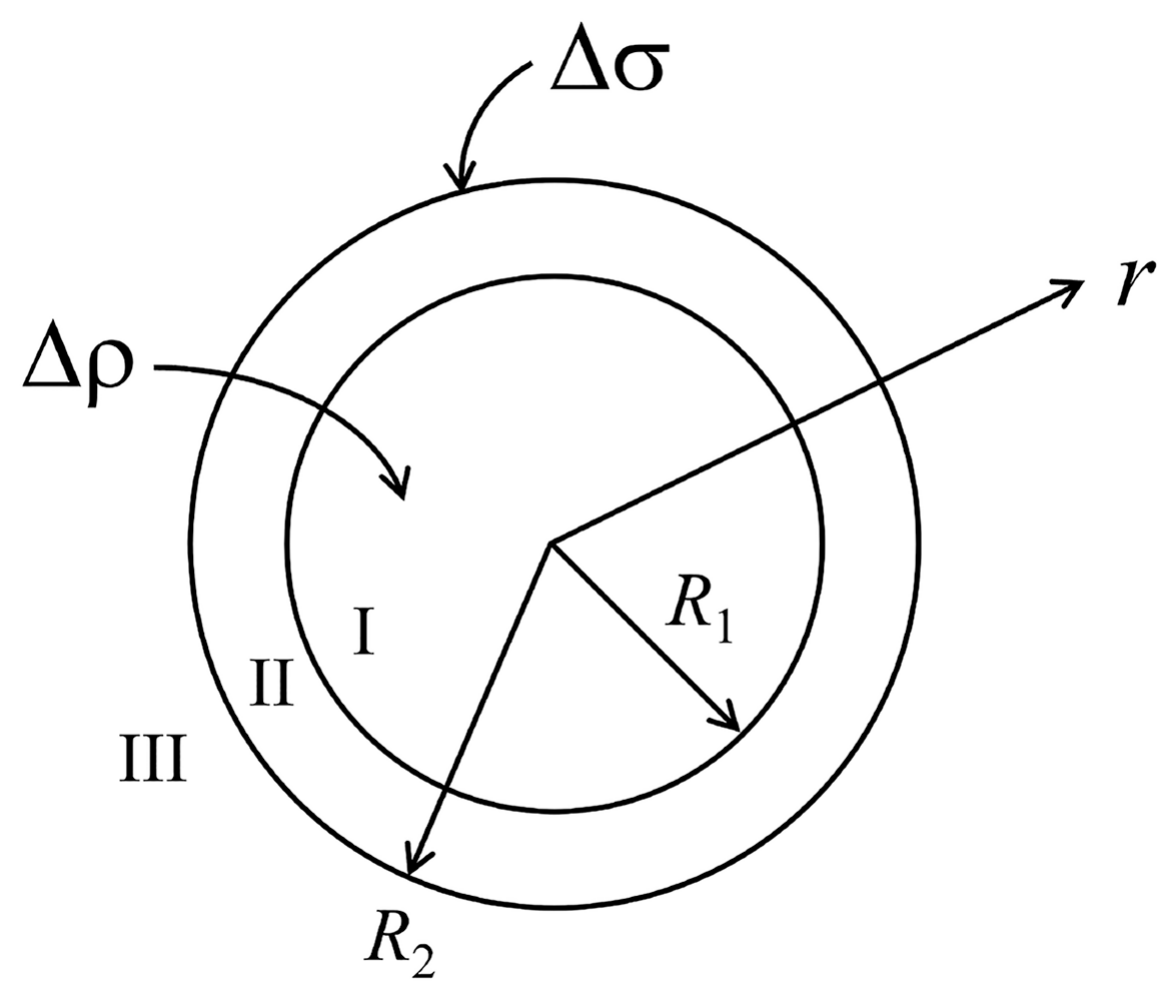
Model of the virus or virus-like particle. Region I is the interior of the protein shell or capsid, for radial distances *r* < *R*_1_. In this region, there is a net immobile space charge of number density Δρ. Region II represents the capsid, with *R*_1_ ≤ *r* ≤ *R*_2_. The outer surface of the capsid is characterized by the presence of a net immobile surface charge of surface number density Δσ. Region III, defined by *r* > *R*_2_, represents the solution outside of the particle. For *r* ≫ *R*_2_, the solution becomes the bulk solution. See also the main text.

To set up the Poisson–Boltzmann theory, we need to construct a model. In our model, we presume that (i) the geometry of the problem obeys spherical symmetry and (ii) all charges associated with the RNA binding domains of the coat proteins and cargo (the “immobile” charges) are uniformly distributed in a spherical volume of radius *R*_1_ > 0. See also Figure 1. So, the number densities  of positive and negative immobile charges are step functions of the radial coordinate *r* ∈ [0, *∞*), with ρ_±_^i^ (as before) their mean densities in the cavity and *H*(*R*_1_ – *r*) = 1 for *r* < *R*_1_ and *H*(*R*_1_ – *r*) = 0 for *r* ≥ *R*_1_ the usual Heaviside step function. The number density associated with the net immobile space charge is defined as . The quantity Δρ ≡ ρ_+_^i^ – ρ_–_^i^ is (as before) the net average number density of immobile charge in the lumen of the particle. It can take both positive and negative values or be equal to zero.

Surrounding the spherical volume is a shell of radius *R*_2_ ≥ *R*_1_. This shell is permeable to mobile ions due to the presence of holes that we do not explicitly model. So, we presume the shell to not contain any mobile or immobile charges, again for reasons of simplicity. On the outer surface of the shell, for *r* = *R*_2_, we envisage the presence of a net number surface density of immobile charges Δσ, noting that this quantity can take positive and negative values or be equal to zero, depending on the type of virus and acidity of the solution. Our model resembles that of Duval and co-workers describing the electrokinetics of the bacteriophage MS2 and end-carboxylated dendrimers.^50,51^ We also note that any transient exposure of the RNA binding domains and cargo to the outside of the capsid shell,^52^ e.g., via the pores, only renormalizes the charge densities of the lumen and the outer surface.

Using the known p*K*_a_s of basic and acidic residues of surface-exposed amino acids, Lošdorfer Božič and collaborators predict that for a large number of viruses −0.3 ≲Δσ ≲ +0.1 nm^–2^ at neutral pH albeit that for the vast majority −0.2 ≲Δσ ≲ 0 nm^–2^.^42^ The predicted net negative charge tallies with the mean isoelectric point of 5.0 ± 1.3 for a large collection of animal, bacterial, and plant viruses.^53^ Available experimental data for the surface charge densities of ϕ29 virion, the ϕ29 prohead, the adenovirus and the minute virus of mice, deduced from atomic force measurements, put Δσ between about 0.01 and 0.4 negative charges per nm^–2^ at near-neutral pH and ionic strengths of 2 and 10 mM.^54^ For CCMV, electrophoretic mobility measurements, and application of the Henry equation, reveal net surface charge number densities between Δσ ≃ – 0.1 nm^–2^ and Δσ ≃ + 0.03 nm^–2^ in the pH range from 3 to 7.5.^55,56^ Finally, entering the bulk solution for *r* > *R*_2_ only mobile ions are present in the solution. The mobile ions in the cavity, for *r* < *R*_1_, and those in the bulk solution, for *r* > *R*_2_, are presumed to be in thermodynamic equilibrium with each other: ions can freely diffuse in and out of the lumen yet we presume that their concentration in the shell region II remains negligible, as already mentioned.^25^

It turns out convenient to work in dimensionless quantities. For that purpose, we define the Debye screening length  in terms of the Bjerrum length λ_B_ and the number density of monovalent salt ρ_s_. The Bjerrum length is defined as λ_B_ = β*e*^2^/4πϵ, with β = 1/*k*_B_*T* being the reciprocal thermal energy, *e* representing the elementary charge, and ϵ the dielectric permittivity of the aqueous medium. As usual, *k*_B_ denotes the Boltzmann constant and *T* the absolute temperature. For room temperature in an aqueous solvent, λ_B_ = 0.71 nm and  nm, with *c*_s_ representing the ionic strength of the added monovalent salt in M. All lengths in our problem shall from this point on be presumed to be scaled by the Debye length, so *r* ≡ *r*/λ_D_, *R*_1_ ≡ *R*_1_/λ_D_ and *R*_2_ ≡ *R*_2_/λ_D_. The electrostatic potential ψ(*r*) we render dimensionless by defining a Coulomb energy scaled to the thermal energy, so ψ ≡ ψ*e*β. For the surface charge density we write Δσ ≡Δσλ_B_λ_D_, and the magnitude of overcharging we capture in the quantity γ ≡Δρ/2ρ_s_. See also the Introduction.

If we insert the Boltzmann distribution, ρ_±_^m^(*r*) = ρ_s_ exp(∓ψ(*r*)), in the dimension-bearing Poisson equation, which reads , we obtain the following Poisson–Boltzmann equation in dimensionless form
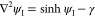
3for the (dimensionless) potential ψ_I_ = ψ in region I, so for radial distances 0 ≤ *r* < *R*_1_,
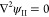
4for the potential ψ_II_ in region II where *R*_1_ ≤ *r* < *R*_2_, and
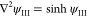
5for the potential ψ_III_ in region III, where *r* ≥ *R*_2_, where we refer again to Figure 1. These equations have to be supplemented with boundary conditions that enforce overall charge neutrality, radial symmetry, and continuity of the potential for all radial positions *r*: (i) ; (ii) ; (iii) ; (iv) ; (v) ; (vi) lim_*r*→*∞*_ψ_III_ = 0.

We have not been able to solve the above set of differential equations exactly. Hence, we follow the prescription of Lifson,^34^ who approximately solved the Poisson–Boltzmann equations for the related problem of a model polyelectrolyte chain in which region II is absent as is the surface charge. (A similar approach in a slightly different geometry was used, e.g., by Ohshima.^48^) Hence, we write ψ_I_(*r*) = ϕ + Δψ_I_(*r*) for 0 ≤ *r* < *R*_1_, where ϕ is a constant background potential and Δψ_I_(*r*) a position-dependent correction to that constant background potential. If we presume that |Δψ_I_| ≪ |ϕ|, we can Taylor expand sinh ψ_I_ = sinh(ϕ + Δψ_I_) = sinh ϕ + cosh ϕ × Δψ_I_(*r*) + ···. If we insert this in eq 3 and realize that the function Δψ_I_ is by construction a varying function of *r*, we obtain the identity sinh ϕ = γ for the constant background potential, and
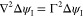
6for the spatially varying part, where
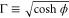
7

It transpires that ϕ must indeed be the Donnan potential, as we find it to obey eq 1 and is part of the solution to the full, nonlinear Poisson–Boltzmann theory in region I. This illustrates once again the deep connection between Donnan theory and Poisson–Boltzmann theory.^34,57^ The correction Δψ_I_ arises in essence because local charge neutrality is broken: mobile ions spread out of the lumen into the bulk solution for reasons of entropy gain. Obviously, the eq 6 that describes this effect can only be accurate under conditions where the linearization holds. Below we evaluate in more detail the conditions under which that this is the case.

Since the potential decays significantly in the intermediate region II, we apply the Debye–Hückel approximation in region III and linearize eq 5, to obtain
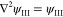
8By comparing eq 6 with eq 8, we are able to conclude that the Debye length in region I must be a factor  smaller than that in the outer region III.^34^ This enhanced electrostatic screening is stronger the larger the magnitude of the Donnan potential. Indeed, for Donnan potentials stronger than the thermal energy, so for |ϕ| ≳ 1, we have , implying that in that case the effective Debye length in region I, λ_D_/Γ, scales as . The magnitude of this effective Debye length is then determined by the mismatch between the number of immobile positive and negative charges in the lumen, and must then be virtually independent of the concentration of salt in the bulk solution.

The remaining set of equations, eqs 4, 6, and 8 can now straightforwardly be solved in polar coordinates and using the boundary conditions quoted above in order to fix all the integration constants. For the dimensionless potential in region I, we find
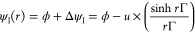
9with
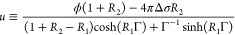
10a parameter that depends on the inner and outer radii of the protein shell *R*_1_ and *R*_2_, the degree of overcharging via the Donnan potential ϕ and the net surface charge density Δσ. Clearly, if |*u* sinh(*R*_1_Γ)/*R*_1_Γ| ≪ |ϕ|, then ψ_I_ ∼ ϕ for *r* < *R*_1_, and the Donnan potential provides a good representation of the electrical potential in region I, the lumen of the protein shell.

The potential in the protein shell (region II) obeys
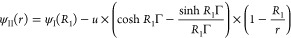
11which is not uniform, unless *u* = 0. This happens exactly if ϕ = 4πΔσ*R*_2_/(1 + *R*_2_), in which case the uniform Donnan potential extends all the way to the boundary with region III, the outer surface of the shell. The potential in region III decays with increasing radial distance *r* under all circumstances, and reads
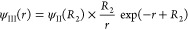
12The somewhat unwieldy expressions, eqs 9–12, agree with those of Lifson for the corresponding case *R*_1_ = *R*_2_ and Δσ = 0.^34^ Lifson found excellent agreement with a numerical solution of the equations for all cases investigated, that is, for fixed γ = 5 and varying *R*_1_ > 1, and for fixed *R*_1_ = 5 and varying γ ≥ 0.5.

To calculate the local pH and relate that to the value in the bulk solution for *r* → *∞*, we again make use of the Boltzmann distribution. Let [H^+^](*r*) be local concentration of H^+^ ions, and  that of the bulk solution. The local concentration of H^+^ ions depends on the local potential ψ via the relation . Hence, the pH depends on the radial coordinate and becomes only equal to the bulk value if ψ → 0, so for *r* → *∞*. Focusing attention on the interior of the capsid, region I, we must have a local concentration of . The average concentration of H^+^ ions in region I, , is equal to
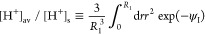
13in terms of the scaled variables *r*, *R*_1_, and ψ_I_. Note that eq 13 applies to any positively charged mobile ionic species, while for negatively charged ionic species, we only need to replace the minus sign in the exponential by a plus sign. Finally, the pH differential between the interior of the capsid and the bulk solution is given by
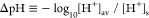
14which we can calculate once the potential in the cavity is known, ψ_I_. Notice that for |*u*| → 0, we have *J* = 1 and we retrieve eq 2 noting that log_10_*e* = 1/ ln 10 with *e* being Euler’s number.

To calculate the pH shift, ΔpH, we insert eq 9 into eq 13, and by applying a suitable change of variables we find from eq 14
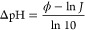
15with
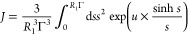
16We have not been able to exactly solve this integral. We expect that under most experimentally relevant conditions |*u* sinh(*R*_1_Γ)/*R*_1_Γ| ≲ 1, allowing us to Taylor expand the exponential and obtain a second order in the parameter *u*,
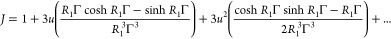
17In the limit *R*_1_Γ ≪ 1, the corrections in powers of *u* are small only if |ϕ – 4πΔσ*R*_2_| ≪ 1. For *R*_1_Γ ≳ 1, they are of the order  implying that as long as the radius of the lumen is much larger than the effective screening length in it, the pH shift should be dominated by the Donnan potential. In the opposite limit, |*u* sinh(*R*_1_Γ)/*R*_1_Γ| ≫ 1, the perturbation approach that we invoke to solve the Poisson–Boltzmann equation in region I should break down. The reason is that in that case |Δψ_I_| is not necessarily small compared to |ϕ| near the edge of the lumen. Hence, we do not discuss this limit any further.

## Results

To investigate in more detail the limits of applicability of Donnan theory, we compare its predictions with our perturbation theory. From eq 9 we read off that provided |*u* sinh(*R*_1_Γ)/*R*_1_Γ| ≪ |ϕ| we have ψ_I_(*r*) ≃ ϕ for all 0 ≤ *r* ≤ *R*_1_, implying that in that case the Donnan potential accurately describes the electrical potential in the lumen of the capsid shell. This happens if at least one of two conditions is met:

(i) The presence of a net charge on the outer surface of the shell compensates for the drop in the potential in the shell, so if ϕ = sinh^–1^γ ≃ 4πΔσ*R*_2_/(1 + *R*_2_). In that case, we have ψ_I_ ≃ ψ_II_ ≃ ϕ, and most of the drop of the potential happens outside of the shell, that is, in region III. This shows that a net surface charge, if not too large and of the same sign as the net immobile space charge present in the lumen, makes the prediction of Donnan theory more accurate than without it.
(ii) If the concentration of salt or the magnitude of the Donnan potential is sufficiently large so that the effective screening length of the solution in the cavity is much smaller than the radius of the lumen, that is, if . For weak degrees of over- or undercharging |γ|≪ 1, this implies that *R*_1_ ≫ 1, while for large degrees of overcharging |γ|≫ 1, the dimensionless radius  may actually be substantially smaller than unity.

From this we conclude that in the present context Donnan theory has a wider range of applicability than is sometimes thought. It is neither restricted to low Donnan potentials^58^ nor to low ionic strengths,^59^ as in fact is already clear from the early work of Lifson.^34^ It seems that modeling a continuous immobile space charge distribution by a series of fixed, localized charges, as was done by Grodzinsky et al.^58^ and Huster et al.,^59^ would lead us to underestimate the range of applicability of Donnan theory.

We now illustrate our findings by taking as model parameters those that we estimate for the *T* = 1 virus-like particles investigated by Maassen and collaborators.^27^ We recall that these are formed by the spontaneous coassembly of coat proteins of CCMV and poly(styrenesulfonate) copolymers. We set the dimensionless shell radii equal to *R*_1_ = 6.5 and *R*_2_ = 12, given the experimental ionic strength of 0.15 M and our estimates for the inner and outer radii of the shell for which we take 5 and 9.5 nm, respectively. The total number of positive charges on the RNA binding domains equals +600. For pHs in the range from 3 and 7.5, around the isoelectric point pI ≃ 4 of the wild-type CCMV virus particles, electrophoretic mobility measurements reveal net surface charge number densities between Δσ ≃ −0.1 nm^–2^ and Δσ ≃ +0.03 nm^–2^.^55^ From this we conclude that the corresponding dimensionless values Δσ must be in the range from −0.055 to +0.016. To produce a pH shift of −0.4 at pHs larger than 6 found experimentally, we obtain from eq 15 a value of ϕ = −0.94 if we set Δσ = −0.055 that should be accurate for pHs larger than 6. This gives γ = −1.08 for the degree of overcharging. These findings are very close to the values of ϕ = −0.92 and γ = −1.06 that we obtain from the Donnan theory for the same system.

**Figure 2:**
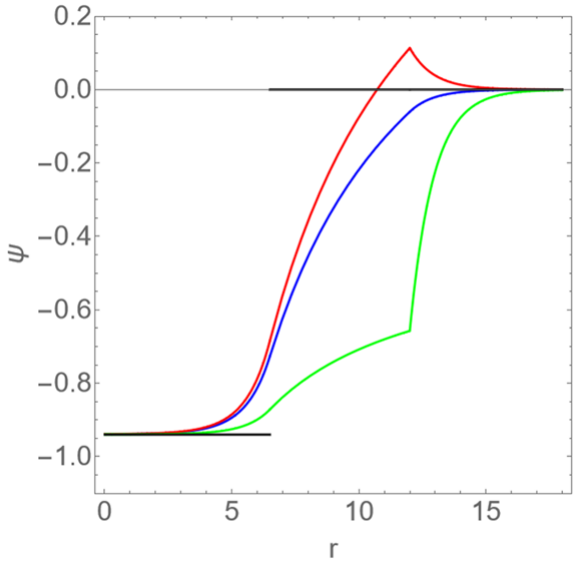
Dimensionless potential ψ as a function of the dimensionless radial distance *r* from the center of a *T* = 1 virus-like particle under conditions of 0.15 M ionic strength. The dimensionless radius of the lumen is *R*_1_ = 6.5 and that of the shell *R*_2_ = 12. From bottom to top: predictions from Poisson–Boltzmann theory with dimensionless surface charge densities Δσ = −0.055, 0, +0.016. The step function represents the Donnan potential ϕ = −0.94 that gives the corresponding average pH difference between lumen and bulk of −0.4.^27^ See also the main text.

Figure 2 shows the dimensionless potential ψ as a function of the dimensionless radial coordinate *r* for the dimensionless surface charge densities Δσ = −0.055, 0, + 0.016. Notice the discontinuity in the radial derivative of the potential for *r* = *R*_2_ for Δσ ≠ 0. Also indicated is the Donnan potential, which is nonzero only in region I, for *r* < *R*_1_ = 6.5. The figure confirms that the Donnan potential reasonably accurately describes the potential in the cavity of the shell for the range of surface charge densities indicated. Only very near the inner surface of the shell the Donnan potential and the potential obtained from Poisson–Boltzmann theory deviate from each other. The discrepancy is particularly small (under ten percent) if the outer surface is negatively charged. For the vast majority of viruses this seems to be the case under conditions of neutral pH.^53^ In view of the above, the agreement is not entirely unexpected, of course, as for the solution conditions of the experiments of Maassen et al. the value of *R*_1_Γ ≃ 7.8 is quite larger than unity.^27^

**Figure 3:**
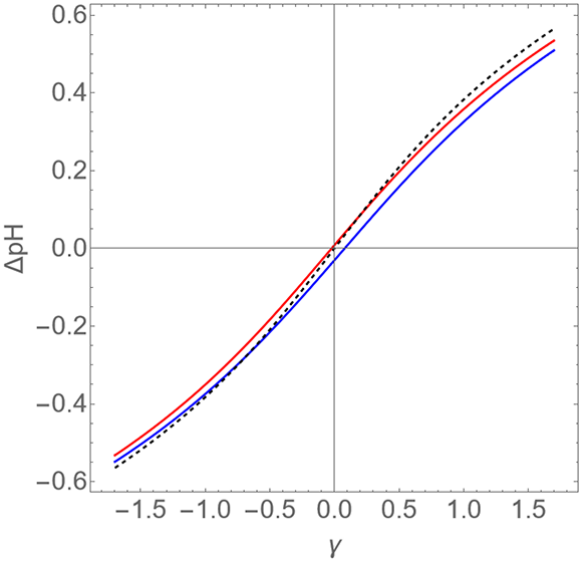
Difference ΔpH between the average pH in the lumen of a *T* = 1 virus-like particle and the pH of the bulk solution as a function of a measure of the imbalance of the number of immobile positive and negative space charges γ in the lumen of the capsid. The dimensionless radius of the lumen is *R*_1_ = 6.5 and that of the shell *R*_2_ = 12 at the fixed ionic strength of 0.15 M. Dashed curve: prediction from Donnan theory. Drawn curves: Δσ = −0.055 (in blue, bottom) and Δσ = +0.016 (in red, top).

Figure 3 shows the predicted pH shift ΔpH as a function of the degree of over- or undercharging γ for dimensionless surface charge densities of Δσ = −0.055 and +0.016 and compares these with the predictions of Donnan theory for the *T* = 1 virus-like particles with *R*_1_ = 6.5 and *R*_2_ = 12 for the fixed ionic strength of 0.15 M, mimicking the experiments of Maassen and collaborators^27^ but now allowing for a variable overcharging. Agreement between our Poisson–Boltzmann theory and Donnan theory is indeed rather good. The figure confirms that the accuracy of Donnan theory increases if the net surface charge has the same sign as the net immobile space charge in the lumen. Finally, Donnan theory slightly overestimates |ΔpH| for larger positive or negative values of γ. This we attribute to the Debye–Hückel approximation becoming less accurate in the outer region III for relatively large degrees of over- or undercharging.

The question arises what happens to the usefulness of Donnan theory if the ionic strength were a factor of, say, ten lower, so 15 mM instead of 150 mM. We would then have *R*_1_ ≃ 2.1 and *R*_2_ ≃ 3.8, which could indicate that Donnan theory might in that case be not quite as accurate. However, if we assume the overcharging to remain more or less constant, then the degree of overcharging becomes much more negative with γ = −11. In that case, we find ϕ = −3.1 and Γ = 3.3. This means that for a ten times smaller ionic strength we have *R*_1_Γ = 6.9, implying that Donnan theory should remain to be reasonable accurate. Indeed, the pH shift predicted by Donnan theory amounts to −1.3 and that from our Poisson–Boltzmann theory is also −1.3, presuming a dimensionless surface charge density of −0.055. We conclude that an increase in the bulk screening length, which in principle would make the Donnan theory less accurate, is more than compensated for by an increase in the magnitude of the Donnan potential that decreases the effective Debye length in the lumen of the virus-like particles.

Let us now apply our theory to the experiments of Ren and collaborators, who encapsulated poly(styrene sulfonic acid) PSA of varying molecular weights from 13 to 990 kDa using the coat proteins of horseradish chlorotic ringspot virus or HCRSV, and predict the pH shift.^60^ PSA is a strong polyacid with a p*K*_a_ ≃ 1, and is fully charged at the pH of about five at which the experiments were done. Ren and collaborators found a more or less constant loading of PSA irrespective of the molecular weight of the PSA, corresponding to a single molecule of 990 kDa. This translates to an encapsulation of 5400 negative charges. The total number of positive charges on the arginine-rich motifs of the *T* = 3 virus amounts to 2880, suggesting an overcharging of about a hundred percent.^61^ At an ionic strength of 0.16 M, and taking for the radius of the lumen 9 nm and that of the shell 15 nm, we get for the scaled dimensions of the shell *R*_1_ = 12 and *R*_2_ = 20.

**Figure 4:**
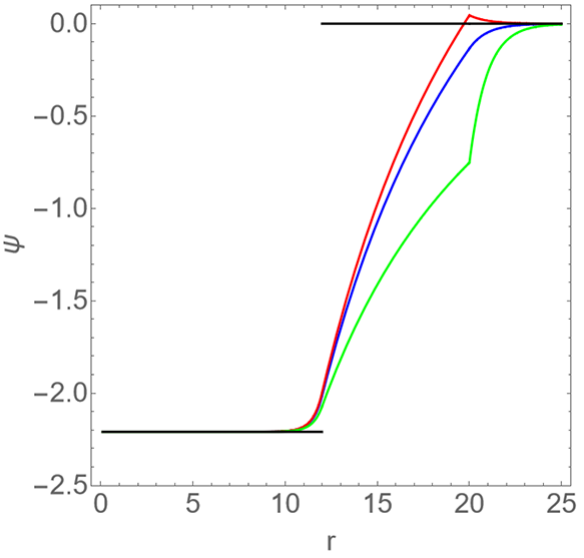
Dimensionless potential ψ as a function of the dimensionless radial distance *r* from the center of a *T* = 3 virus-like particle under conditions of 0.16 M ionic strength, 2880 positive charges on the RNA binding domains and 5400 negative charges on the encapsulated polyanion mimicking the experiments of Ren and collaborators.^60^ For the dimensionless radius of the lumen we set *R*_1_ = 12 and for that of the shell *R*_2_ = 20. From bottom to top predictions from Poisson–Boltzmann theory with dimensionless surface charge densities Δσ = −0.055, 0, +0.016. The step function represents the Donnan potential ϕ. See also the main text.

Hence, our measure for the degree of overcharging attains the value of γ = −4.3, the corresponding Donnan potential ϕ = −2.2 and the reduction factor of the Debye length Γ = 2.1. We conclude that Donnan theory should be rather accurate, as Figure 4 in fact confirms for −0.055 ≤ Δσ ≤ +0.016. Notice that the so-called zeta potential of the virus-like particles measured by Ren et al. has values between between −2.3 and −2.9 mV,^60^ which translates to a net surface charge number density of about −0.016 nm^–2^ if we apply the Henry equation.^62^ Application of the Henry equation is justified, provided capsids are impermeable to the flow field. This seems to be the case for capsids of simple plant viruses^56^ albeit that it need not be generally true for all types of particle.^49,50^ Presuming the quoted value for the surface charge density to be accurate, the corresponding dimensionless surface charge density becomes Δσ ≃ −8.5 × 10^–3^ for the given ionic strength of the solution. The pH shifts we then obtain from Donnan theory and from Poisson–Boltzmann theory are for all intents and purposes equal, namely ΔpH = −0.94.

## Discussion and Conclusions

An imbalance in the number of localized positive and negative space charges in the lumina of viruses, virus-like particles and other types of protein shell, semipermeable to mobile ionic species, gives rise to a Donnan potential difference between the bulk solution and the inside of the particles. This potential difference induces a pH differential that can potentially be large. This, in itself, is not surprising.^32^ What is surprising, given the small size of the particles, is that simple Donnan theory is remarkably accurate in predicting the magnitude of this pH shift when compared with predictions from Poisson–Boltzmann theory, as it presumes local charge neutrality and homogeneous distributions of mobile ions in- and outside the shell. Under typical experimental conditions, the presence of a net charge on the outer surface of the shell turns out to have a relatively minor effect. For overcharged virus-like particles the presence of a negative surface charge actually makes Donnan theory more rather than less accurate.

The reason why Donnan theory works so well is that, in practice, the Donnan potential in the lumina of the particles is sufficiently large so that the effective (local) screening length in it becomes small on the scale of the width of the lumen the region in it where most of the immobile charges can be found. A similar conclusion can be drawn for other types of particle characterized by an immobile space charge distribution.^34,48,51^ Consequently, even for the smallest of virus-like particles the concentration of mobile ions remains more or less constant in the 10 nm or so wide lumen, at least if the immobile space charge distribution is also more or less uniform. Even if the immobile charges are not uniformly distributed in the lumen but localized in a region near the inner surface of the shell, we would still expect the predictions for the pH shift in that region to be well-described by Donnan theory as long as it is (much) wider than the effective screening length.^50^

All of this implies that simple Donnan theory can indeed be used to estimate the total amount of encapsulated material inside of small proteinaceous shells from the measured pH, as was done by Maassen et al. for virus-like particles self-assembled from the coat proteins of the plant virus CCMV and a polyanionic cargo.^27^ Conversely, if the amount of encapsulated polyanionic material is known, such as in the experiments of Gelbart et al.^8,10^ and of Lim et al.,^60^ Donnan theory can be used to estimate the pH in the lumen of the virus-like particle. As discussed in the Introduction, the predicted pH shifts can be as large as a full pH unit, in both positive and negative directions. If virus-like particles are used for catalysis, e.g., by way of encapsulated enzymes, then the activity of these enzymes may be influenced not only by the compartmentalization itself but also by the local pH and ionic strength.^63,64^ Since the charged state of enzymes depends on the pH, they would modify the Donnan potential self-consistently if their number is large enough. In fact, the coat protein itself might be involved in buffering activity.^27^ This kind of charge regulation can be incorporated in both Donnan and Poisson–Boltzmann theory relatively straightforwardly.^35,50,65^

For instance, if the cargo is a weak polyacid, such as poly(acrylic acid) ,^60^ we only need to modify the parameter , which now becomes a function of the local potential ψ_I_ in the lumen, for
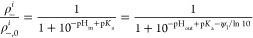
18This, of course, is the familiar Henderson–Hasselbalch equation, where in the second equality we have expressed the local pH in the lumen, pH_in_, in terms of the pH in the bulk solution, pH_out_. Further, p*K*_a_ is the dissociation constant of the weak polyacid, and ρ_–,0_^*i*^ the concentration of chargeable groups on the encapsulated polyacid that we again presume to be uniformly distributed in the lumen. At the level of Donnan theory, ψ_I_ = ϕ, while within our Poisson–Boltzmann theory, we would write again ψ_I_ = ϕ + Δψ_I_ and expand sinh ψ_I_ as well as γ to first order in Δψ_I_. The Debye length renormalization factor Γ now obeys the equality . As long as *R*_1_Γ remains sufficiently large, the Donnan theory should again be reasonably accurate.

Equation 18 tells us that the pH shift ΔpH now depends on the pH of the bulk solution pH_out_, except if |pH_in_ – p*K*_a_| ≳ 1. For |pH_in_ – p*K*_a_| ≲ 1, the pH shift can actually compensate for changes in the outside pH, leading to a constant pH inside the particle. This happens if ∂ΔpH/∂pH_out_ = −1, which within Donnan theory translates to ∂ϕ/∂pH_out_ = – ln 10. It shows that charge regulation can indeed lead to a buffering effect, explaining the findings of Maassen and collaborators, who find a constant (negative) pH shift for solution pHs from 6 to 8 but a constant inside pH for solution pHs between 5 and 6.^27^ In their particular case, the buffering is arguably not caused by the encapsulated strong polyanion, but by moieties on the coat protein itself.

We shall not dwell on this issue any further and end by mentioning that the calculation involving charge regulation would allow us to establish the mean charge on any encapsulated weak polyacid and compare that with the mean charge of the same polyacid in free solution. This, with the aforementioned experiments of Lim and collaborators in mind.^60^ Note that the charged state of the weak polyacids in free solution and that in the capsids are in principle not the same due to the impact of what essentially is the Donnan potential.^13^ We leave this for future work.

## References

[ref1] McPherson A. Micelle formation and crystallization as paradigms for virus assembly. BioEssays 2005, 27, 447–458. 10.1002/bies.20196. 15770675

[ref2] Buzón P.; Maity S.; Roos W. H. Physical virology: from virus self-assembly to particle mechanics. WIREs Nanomed. Nanobiotechnol. 2020, 12, e1613 10.1002/wnan.1613. PMC7317356 31960585

[ref3] Garmann R. F.; Comas-Garcia M.; Knobler C. M.; Gelbart W. M. Physical principles in the self-assembly of a simple spherical virus. Acc. Chem. Res. 2016, 49, 48–55. 10.1021/acs.accounts.5b00350. 26653769

[ref4] Ni P.; Wang Z.; Ma X.; Das N. C.; Sokol P.; Chiu W.; Dragnea B.; Hagan M.; Kao C. C. An examination of the electrostatic interactions between the N-terminal tail of the Brome Mosaic Virus coat protein and encapsidated RNAs. J. Mol. Biol. 2012, 419, 284–300. 10.1016/j.jmb.2012.03.023. 22472420 PMC3360812

[ref5] Bancroft J. B.; Hiebert E.; Bracker C. E. The effects of various polyanions on shell formation of some spherical viruses. Virology 1969, 39, 924–930. 10.1016/0042-6822(69)90029-4. 5358835

[ref6] Fox J. M.; Wang G.; Speir J. A.; Olson N. H.; Johnson J. E.; Baker T. S.; Young M. J. Comparison of the native CCMV virion with in vitro assembled CCMV virions by cryoelectron microscopy and image reconstruction. Virology 1998, 244, 212–218. 10.1006/viro.1998.9107. 9581792

[ref7] Sun J.; DuFort C.; Daniel M.-C.; Murali A.; Chen C.; Gopinath K.; Stein B.; De M.; Rotello V. M.; Holzenburg A.; Kao C. C.; Dragnea B. Core-controlled polymorphism in virus-like particles. Proc. Natl. Acad. Sci. U.S.A. 2007, 104, 1354–1359. 10.1073/pnas.0610542104. 17227841 PMC1783121

[ref8] Hu Y.; Zandi R.; Anavitarte A.; Knobler C. M.; Gelbart W. M. Packaging of a polymer by a viral capsid: the interplay between polymer length and capsid size. Biophys. J. 2008, 94, 1428–1436. 10.1529/biophysj.107.117473. 17981893 PMC2212672

[ref9] de la Escosura A.; Janssen P. G. A.; Schenning A. P. H. J.; Nolte R. J. M.; Cornelissen J. J. L. M. Encapsulation of DNA-templated chromophore assemblies within virus protein nanotubes. Angew. Chem., Int. Ed. 2010, 49, 5335–5338. 10.1002/anie.201001702. 20803703

[ref10] Cadena-Nava R. D.; Hu Y.; Garmann R. F.; Ng B.; Zelikin A. N.; Knobler C. M.; Gelbart W. M. Exploiting fluorescent polymers to probe the self-assembly of virus-like particles. J. Phys. Chem. B 2011, 115, 2386–2391. 10.1021/jp1094118. 21338131

[ref11] de Ruiter M. V.; van der Hee R. M.; Driessen A. J. M.; Keurhorst E. D.; Hamid M.; Cornelissen J. J. L. M. Polymorphic assembly of virus-capsid proteins around DNA and the cellular uptake of the resulting particles. J. Controlled Release 2019, 307, 342–354. 10.1016/j.jconrel.2019.06.019. 31228473

[ref12] Zandi R.; van der Schoot P. Size Regulation of ss-RNA Virusess. Biophys. J. 2009, 96, 9–20. 10.1529/biophysj.108.137489. 18931258 PMC2710049

[ref13] Kusters R.; Lin H.-K.; Zandi R.; Tsvetkova I.; Dragnea B.; van der Schoot P. Role of charge regulation and size polydispersity in nanoparticle encapsulation by viral coat proteins. J. Phys. Chem. B 2015, 119, 1869–1880. 10.1021/jp5108125. 25562399

[ref14] Ceres P.; Zlotnick A. Weak protein-protein interactions are sufficient to drive assembly of Hepatitis B Virus capsids. Biochemistry 2002, 41, 11525–11531. 10.1021/bi0261645. 12269796

[ref15] Lavelle L.; Gingery M.; Phillips M.; Gelbart W. M.; Knobler C. M.; Cadena-Nava R. D.; Vega-Acosta J. R.; Pinedo-Torres L. A.; Ruiz-Garcia J. Phase diagram of self-assembled viral capsid protein polymorphs. J. Phys. Chem. B 2009, 113, 3813–3819. 10.1021/jp8079765. 19673134

[ref16] Timmermans S. B. P. E.; Ramezani A.; Montalvo T.; Nguyen M.; van der Schoot P.; van Hest J. C. M.; Zandi R. The dynamics of viruslike capsid assembly and disassembly. J. Am. Chem. Soc. 2022, 144, 12608–12612. 10.1021/jacs.2c04074. 35792573 PMC9305980

[ref17] Kegel W. K.; van der Schoot P. Competing hydrophobic and screened-Coulomb interactions in Hepatitis B Virus capsid assembly. Biophys. J. 2004, 86, 3905–3913. 10.1529/biophysj.104.040055. 15189887 PMC1304292

[ref18] Zandi R.; Dragnea B.; Travesset A.; Podgornik R. On virus growth and form. Phys. Rep. 2020, 847, 1–102. 10.1016/j.physrep.2019.12.005.

[ref19] Ma Y.; Nolte R. J. M.; Cornelissen J. J. L. M. Virus-based nanocarriers for drug delivery. Adv. Drug Delivery Rev. 2012, 64, 811–825. 10.1016/j.addr.2012.01.005. 22285585

[ref20] Wen A. M.; Podgornik R.; Strangi G.; Steinmetz N. F. Photonics and plasmonics go viral: self-assembly of hierarchical metamaterials. Rend. Ris. Acc. Lincei 2015, 26, 129–141. 10.1007/s12210-015-0396-3. PMC5509229 28713533

[ref21] Wen A. M.; Steinmetz N. F. Design of virus-based nanomaterials for medicine, biotechnology, and energy. Chem. Soc. Rev. 2016, 45, 4074–4126. 10.1039/C5CS00287G. 27152673 PMC5068136

[ref22] McNeale D.; Dashti N.; Cheah L. C.; Sainsbury F. Protein cargo encapsulation by virus-like particles: Strategies and applications. WIREs Nanomed. Nanobiotechnol. 2022, e1869 10.1002/wnan.1869. 36345849

[ref23] Oerlemans R. A. J. F.; Timmermans S. B. P. E.; Hest J. C. M. Artificial organelles: towards adding or restoring intracellular activity. ChemBioChem 2021, 22, 2051–2078. 10.1002/cbic.202000850. 33450141 PMC8252369

[ref24] Selivanovitch E.; LaFrance B.; Douglas T. Molecular exclusion limits for diffusion across a porous capsid. Nat. Commun. 2021, 12, 2903 10.1038/s41467-021-23200-1. 34006828 PMC8131759

[ref25] Durham A. C. H.; Witz J.; Bancroft J. B. The semipermeability of simple spherical virus capsids. Virology 1984, 133, 1–8. 10.1016/0042-6822(84)90419-7. 18639804

[ref26] Uchida M.; Manzo E.; Echeveria D.; Jiménez S.; Lovell L. Harnessing physicochemical properties of virus capsids for designing enzyme confined nanocompartments. Curr. Opin. Virol. 2022, 52, 250–257. 10.1016/j.coviro.2021.12.012. 34974380 PMC8939255

[ref27] Maassen S. J.; van der Schoot P.; Cornelissen J. J. L. M. Experimental and theoretical determination of the pH inside the confinement of a virus-like particle. Small 2018, 14, 1802081 10.1002/smll.201802081. 30102454

[ref28] Huang J.; Jiang Q.; Yang M.; Dykes G. F.; Weetman S. L.; Xin W.; He H.-L.; Liu L.-N. Probing the internal pH and permeability of a carboxysome shell. Biomacromolecules 2022, 23, 4339–4348. 10.1021/acs.biomac.2c00781. 36054822 PMC9554877

[ref29] Philipse A.; Vrij A. The Donnan equilibrium: I. On the thermodynamic foundation of the Donnan equation of state. J. Phys.: Condens. Matter 2011, 23, 194106 10.1088/0953-8984/23/19/194106. 21525564

[ref30] Langridge-Smith J. E.; Dubinsky W. P. Donnan equilibrium and pH gradient in isolated tracheal apical membrane vesicles. Am. J. Physiol. 1985, 249, C417–C420. 10.1152/ajpcell.1985.249.5.C417. 4061628

[ref31] Donnan F. G. Theorie der Membrangleichgewichte und Membranpotentiale bei Vorhandensein von nicht dialysierenden Elektrolyten. Ein Beitrag zur physikalisch-chemischen Physiologie. Z. Elektrochem. Angew. Phys. Chem. 1911, 17, 572–581.

[ref32] Donnan F. G. The theory of membrane equilibria. Chem. Rev. 1924, 1, 73–90. 10.1021/cr60001a003.

[ref33] Kimball G. E.; Cutler M.; Samelson H. A theory for polyelectrolytes. J. Phys. Chem. 1952, 56, 57–60. 10.1021/j150493a012.

[ref34] Lifson S. Improved approximation for the potential of spherical polyelectrolyte molecules in solution. J. Chem. Phys. 1957, 27, 700–701. 10.1063/1.1743817.

[ref35] Haložan D.; Sukhorukov G. B.; Brumen M.; Donath E.; Möhwald H. Donnan equilibrium and osmotic pressure in hollow polyelectrolyte microcapsules. Acta Chim. Slov. 2007, 54, 598–604.

[ref36] Angelescu D. G.; Stenhammar J.; Linse P. Packaging of a flexible polyelectrolyte inside a viral capsid: effect of salt concentration and salt valence. J. Phys. Chem. B 2007, 111, 8477–8485. 10.1021/jp068384o. 17604391

[ref37] van der Schoot P.; Bruinsma R. Electrostatics and the assembly of an RNA virus. Phys. Rev. E 2005, 71, 061928 10.1103/PhysRevE.71.061928. 16089786

[ref38] Belyi V. A.; Muthukumar M. Electrostatic origin of the genome packing in viruses. Proc. Natl. Acad. Sci. U.S.A. 2006, 103, 17174–17178. 10.1073/pnas.0608311103. 17090672 PMC1859905

[ref39] Hu T.; Zhang R.; Shklovskii B. I. Electrostatic theory of viral self-assembly. Physica A 2008, 387, 3059–3064. 10.1016/j.physa.2008.01.010.

[ref40] Ting C. L.; Wu J.; Wang Z.-G. Thermodynamic basis for the genome to capsid charge relationship in viral encapsidation. Proc. Natl. Acad. Sci. U.S.A. 2011, 108, 16986–16991. 10.1073/pnas.1109307108. 21969546 PMC3193252

[ref41] Božič A. L.; Podgornik R. Varieties of charge distributions in coat proteins of ssRNA+ viruses. J. Phys.: Condens. Matter 2018, 30, 024001 10.1088/1361-648X/aa9ded. 29182522 PMC7104810

[ref42] Božič A. L.; Šiber A.; Podgornik R. How simple can a model of an empty viral capsid be? Charge distributions in viral capsids. J. Biol. Phys. 2012, 38, 657–671. 10.1007/s10867-012-9278-4. 24615225 PMC3473132

[ref43] Zandi R.; van der Schoot P. Impact of the topology of viral RNAs on their encapsulation by virus coat proteins. J. Biol. Phys. 2013, 39, 289–299. 10.1007/s10867-013-9307-y. 23860874 PMC3662416

[ref44] Marichal L.; Gargowitsch L.; Rubim R. L.; Sizun C.; Kra K.; Bressanelli S.; Dong Y.; Panahandeh S.; Zandi R.; Tresset G. Relationships between RNA topology and nucleocapsid structure in a model icosahedral virus. Biophys. J. 2021, 120, 3925–3936. 10.1016/j.bpj.2021.08.021. 34418368 PMC8511167

[ref45] Odijk T.; Slok F. Nonuniform Donnan equilibrium within bacteriophages packed with DNA. J. Phys. Chem. B 2003, 107, 8074–8077. 10.1021/jp0224822.

[ref46] Philipse A. Remarks on the Donnan condenser in the sedimentation–diffusion equilibrium of charged colloids. J. Phys.: Condens. Matter 2004, 16, S4051–S4062. 10.1088/0953-8984/16/38/020.

[ref47] Duval J. F. L. Electrokinetics of Diffuse Soft Interfaces. 2. Analysis Based on the Nonlinear Poisson-Boltzmann Equation. Langmuir 2005, 21, 3247–3258. 10.1021/la040108i. 15807561

[ref48] Ohshima H. Donnan potential and surface potential of a spherical soft particle in an electrolyte solution. J. Colloid Interface Sci. 2008, 323, 92–97. 10.1016/j.jcis.2008.03.021. 18417149

[ref49] Gopmandal P. P.; Duval J. F. L. Electrostatics and electrophoresis of engineered nanoparticles and particulate environmental contaminants: Beyond zeta potential-based formulation. Curr. Opin. Coll. Iinterface Sci. 2022, 60, 101605 10.1016/j.cocis.2022.101605.

[ref50] Langlet J.; Gaboriaud F.; Gantzer C.; Duval J. F. L. Impact of chemical and structural anisotropy on the electrophoretic mobility of spherical soft multilayer particles: the case of bacteriophage MS2. Biophys. J. 2008, 94, 3293–3312. 10.1529/biophysj.107.115477. 18192368 PMC2275710

[ref51] Moussa M.; Caillet C.; Town R. M.; Duval J. F. L. Remarkable electrokinetic features of charge-stratified soft nanoparticles: mobility reversal in monovalent aqueous electrolyte. Langmuir 2015, 31, 5656–5666. 10.1021/acs.langmuir.5b01241. 25939023

[ref52] Selzer L.; Kant R.; Wang J. C.-Y.; Bothner B.; Zlotnick A. Hepatitis B Virus core protein phosphorylation sites affect capsid stability and transient exposure of the C-terminal domain. J. Biol. Chem. 2015, 290, 28584–28593. 10.1074/jbc.M115.678441. 26405031 PMC4653712

[ref53] Michen B.; Graule T. Isoelectric points of viruses. J. Appl. Microbiol. 2010, 109, 388–397. 10.1111/j.1365-2672.2010.04663.x. 20102425

[ref54] Hernando-Perez M.; Cartagena-Rivera A. X.; Losdorfer Bozic A.; Carrillo P. J. P.; San Martin C.; Mateu M. G.; Raman A.; Podgornik R.; de Pablo P. J. Quantitative nanoscale electrostatics of viruses. Nanoscale 2015, 7, 17289–17298. 10.1039/C5NR04274G. 26228582

[ref55] Vega-Acosta J. R.; Cadena-Nava R. D.; Gelbart W. M.; Knobler C. M.; Ruiz-García J. Electrophoretic mobilities of a viral capsid, its capsid Protein, and their relation to viral assembly. J. Phys. Chem. B 2014, 118, 1984–1989. 10.1021/jp407379t. 24467401

[ref56] Duran-Meza A. L.; Villagrana-Escareno M. V.; Ruiz-Garcia J.; Knobler C. M.; Gelbart W. M. Controlling the surface charge of simple viruses. PLoS One 2021, 16, e0255820 10.1371/journal.pone.0255820. 34506491 PMC8432797

[ref57] Philipse A. P.; Kuipers B. W. M.; Vrij A. Algebraic repulsions between charged planes with strongly overlapping electrical double layers. Langmuir 2013, 29, 2859–2870. 10.1021/la3049482. 23383698

[ref58] Basser P. J.; Grodzinsky A. J. The Donnan model derived from microstructure. Biophys. Chem. 1993, 46, 57–68. 10.1016/0301-4622(93)87007-J. 8443336

[ref59] Dähnert K.; Huster D. Comparison of the Poisson–Boltzmann Model and the Donnan Equilibrium of a Polyelectrolyte in Salt Solution. J. Colloid Interface Sci. 1999, 215, 131–139. 10.1006/jcis.1999.6238. 10362482

[ref60] Ren Y.; Wong S.-M.; Lim L.-Y. In vitro-reassembled plant virus-like particles for loading of polyacids. J. Gen. Virol. 2006, 87, 2749–2754. 10.1099/vir.0.81944-0. 16894216

[ref61] Doan D. N. P.; Lee K. C.; Laurinmäki P.; Butcher S.; Wong S.-M.; Dokland T. Three-dimensional reconstruction of hibiscus chlorotic ringspot virus. J. Struct. Virol. 2003, 144, 253–261. 10.1016/j.jsb.2003.10.001. 14643194

[ref62] Hunter R. J. Zeta Potential in Colloid Science; Colloid Science; Academic Press: London, United Kingdom, 1981.

[ref63] Minten I. J.; Claessen V. I.; Blank K.; Rowan A. E.; Nolte R. J. M.; Cornelissen J. J. L. M. Catalytic capsids: the art of confinement. Chem. Sci. 2011, 2, 358–362. 10.1039/C0SC00407C.

[ref64] Schoonen L.; Nolte R. J. M.; van Hest J. C. M. Highly efficient enzyme encapsulation in a protein nanocage: towards enzyme catalysis in a cellular nanocompartment mimic. Nanoscale 2016, 8, 14467–14472. 10.1039/C6NR04181G. 27407020

[ref65] Khunpetch P.; Majee A.; Podgornik R. Curvature effects in charge-regulated lipid bilayers. Soft Matt. 2022, 18, 2597–2610. 10.1039/D1SM01665B. 35294512

